# Mechanism of Curcumin in Inhibiting Proliferation of Head and Neck Squamous Cell Carcinoma: A Network Pharmacology and Cellular Experimental Study

**DOI:** 10.1155/bmri/4318115

**Published:** 2025-11-26

**Authors:** Yating He, Yaqi Liao, Shizhen Fang, Ling Zhu, Zhang Zhao, Tingting Chen, Zhimin Zhang

**Affiliations:** ^1^ Department of Otolaryngology, The Third People’s Hospital of Hubei Province, Wuhan, Hubei, China; ^2^ Department the Third People’s Hospital of Hubei Province, Hubei University of Medicine, Shiyan, Hubei, China, hbmu.edu.cn

**Keywords:** curcumin, EGFR/STAT3 signaling pathway, head and neck squamous cell carcinoma, network pharmacology, RNA sequencing

## Abstract

Despite advances in cancer therapy, head and neck squamous cell carcinoma (HNSCC) remains a challenging malignancy with limited treatment options, prompting this investigation into curcumin′s antitumor mechanisms through integrated network pharmacology, molecular docking, and in vitro experiments. Our comprehensive analysis identified 34 potential targets, with AKT1, EGFR, and STAT3 emerging as core targets primarily involved in regulating proliferation, apoptosis, and migration via the EGFR/STAT3 pathway. Experimental validation demonstrated curcumin′s dose‐dependent inhibition of viability, invasion, and migration in FaDu and CAL 27 cells, while promoting apoptosis and downregulating EGFR/STAT3 expression at both mRNA and protein levels—effects that were synergistically enhanced when combined with AG490 inhibitor. RNA‐seq analysis further confirmed STAT pathway suppression as a key anticancer mechanism, collectively establishing curcumin′s therapeutic potential through EGFR/STAT3 axis modulation. Overall, these preliminary network pharmacology and in vitro experimental results suggest that curcumin is a potential therapeutic agent for HNSCC and is worthy of further study. This study provides a certain theoretical basis for future clinical exploration.

## 1. Introduction

The rapid socioeconomic development and environmental changes have positioned cancer as a critical global health challenge, with escalating incidence and mortality rates posing substantial threats to human life. According to the latest Global Cancer Statistics from the International Agency for Research on Cancer (2022), approximately 20 million new cancer cases were reported worldwide, accompanied by 9.7 million cancer‐related deaths, with projections indicating a rise to 35 million new cases by 2050 [1]. Among these, head and neck squamous cell carcinoma (HNSCC), originating from the mucosal epithelium of oral, nasal, pharyngeal, and laryngeal regions, represents over 90% of all head and neck malignancies. Ranked as the sixth most prevalent cancer globally, HNSCC demonstrates a steadily increasing incidence [2].

Current clinical management of HNSCC employs multimodal strategies combining surgical resection and radiotherapy as foundational approaches, supplemented by chemotherapy, targeted agents, and immunotherapy [3]. However, the irreversible iatrogenic damage from radical surgery and radiotherapy, including structural deformities and functional impairments, remains a significant concern. Despite therapeutic advancements in precision medicine over recent decades, the 5‐year survival rate persists below 50%, highlighting limitations in existing treatment paradigms [4]. Global Cancer Observatory data (2018) documented 890,000 new HNSCC cases and 450,000 deaths annually, with projections suggesting a 30% incidence increase to 1.08 million new cases by 2030 [5]. These epidemiological trends underscore the urgent need for developing efficacious, low‐toxicity therapeutic alternatives from medicinal plants or natural products to overcome current therapeutic plateaus.

Traditional Chinese medicine (TCM), embodying millennia of clinical experience, has gained increasing research attention for its anticancer potential. Curcumin, a polyphenolic compound derived from *Curcuma longa* rhizomes, exhibits broad pharmacological properties including antimicrobial, anti‐inflammatory, antioxidant, anticancer, and immunomodulatory effects [6–10]. Extensive preclinical studies and ongoing clinical trials demonstrated its therapeutic potential across diverse pathologies, including Alzheimer’s disease, various cancers, and cardiovascular disorders [11–16]. Mechanistically, curcumin modulates multiple biological targets—transcription factors, enzymatic activities, receptor signaling, and cytokine networks—through specific molecular interactions [17]. This polypharmacological profile enables systemic regulation through interconnected signaling pathways.

The paradigm shift from reductionist to holistic approaches in modern medicine aligns with TCM′s systemic perspective, emphasizing multitarget interventions and network‐based disease modulation [18, 19]. This convergence facilitated the emergence of network pharmacology, a discipline pioneered by Hopkins [20] that integrates multiomics data to elucidate drug–target–disease networks. By transcending the “single‐target” paradigm, network pharmacology provides a framework for understanding how bioactive compounds coordinately regulate disease‐associated protein networks to restore homeostasis [21]. Its applications in drug discovery—particularly for natural products—include target prediction, molecular docking, and experimental validation, offering cost‐effective strategies for mechanistic exploration.

Our study employed this integrative methodology to investigate curcumin′s anti‐HNSCC mechanisms. First, network pharmacology identified core targets and signaling pathways (e.g., EGFR/STAT3), validated through molecular docking. Subsequent in vitro experiments—including CCK‐8 proliferation assays, flow cytometric apoptosis analysis, Transwell migration/invasion tests, western blotting, RT‐qPCR, and RNA‐seq—systematically evaluated curcumin′s effects on HNSCC cell phenotypes and molecular profiles. This multiplatform approach provided comprehensive evidence supporting curcumin′s therapeutic potential against HNSCC through network‐based pharmacological actions.

## 2. Materials and Methods

### 2.1. Network Pharmacology Analysis

#### 2.1.1. Target Prediction

The SMILES structure of curcumin was obtained from the PubChem database and submitted to SwissTargetPrediction (species: *Homo sapiens*) to identify potential targets (probability > 0). HNSCC‐related targets were retrieved from GeneCards (relevance score > 50) and OMIM databases using the keyword “Squamous Cell Carcinoma of Head and Neck”.

#### 2.1.2. Protein–Protein Interaction (PPI) Network Construction

Common targets between curcumin and HNSCC were identified using Venny 2.1 and used to construct a PPI network via the STRING database. The network was analyzed using Cytoscape 3.10.1 with topological parameters (degree, closeness, and betweenness centrality).

#### 2.1.3. Enrichment Analysis

Functional enrichment analysis of GO terms and KEGG pathways was performed using the DAVID database (species: *Homo sapiens*), with the Top 10 GO terms and Top 20 KEGG pathways selected for further investigation.

#### 2.1.4. Molecular Docking

Protein structures of core targets were downloaded from the PDB database. Curcumin and receptor proteins were prepared using AutoDockTools 1.5.7. Docking results were visualized with PyMol software.

### 2.2. Cell Experiments

#### 2.2.1. Preparation of Curcumin

The curcumin used in this study (CAS: 458‐37‐7, purity ≥ 98*%*) was purchased from Macklin Biochemical Co., Ltd. (Shanghai, China). A stock solution of 8 mg/mL was prepared by dissolving curcumin in dimethyl sulfoxide (DMSO) and stored at 4°C. Prior to use, the stock solution was diluted to the desired concentrations with the corresponding culture medium.

#### 2.2.2. Cell Culture

FaDu and CAL 27 cell lines (authenticated by STR profiling) were obtained from Wuhan Linsi Biotechnology Co., Ltd. Cells were maintained in DMEM (Procell) supplemented with 10% FBS (fetal bovine serum, Excell Bio) at 37°C with 5% CO_₂_. Subculture was performed using 0.25% trypsin (Procell).

#### 2.2.3. Cell Viability Assay

Cells were seeded in 96‐well plates (1 × 10^4^ cells/well) and treated with curcumin (0–8 *μ*g/mL) for 48 h. Cell viability was assessed using the CCK‐8 assay (HYCEZMBIO) by measuring absorbance at 450 nm.

#### 2.2.4. Migration and Invasion Assays

For migration assay, cells (4 × 10^5^/mL) were seeded in Transwell chambers (FALCON) without Matrigel coating. For invasion assay, chambers were precoated with Matrigel (5 mg/mL). After 24 h incubation, migrated/invaded cells were stained with crystal violet and quantified.

#### 2.2.5. Apoptosis Analysis

Cell apoptosis was detected using Annexin V‐APC/7‐AAD double staining (KeyGEN BioTECH) followed by flow cytometry analysis (Beckman Coulter).

### 2.3. Molecular Biology Assays

#### 2.3.1. Quantitative Real‐Time PCR (qRT‐PCR)

Total RNA was extracted using TRIzol reagent (Invitrogen) and reverse transcribed into cDNA. Primer sequences are listed in Table 1. Gene expression was analyzed using the 2‐*ΔΔ*Ct method.

**Table 1:** Primer sequences for qRT‐PCR.

**Gene**	**Primer sequence (5** ^′^ **-3** ^′^ **)**
*β*‐Actin‐F	GCACTCTTCCAGCCTTCCTT
*β*‐Actin‐R	TTCATTGTGCTGGGTGCCA
EGFR‐F	GCGCTACCTTGTCATTCAGG
EGFR‐R	TATCAATGCAAGCCACGGTG
STAT3‐F	TCAGTGACCAGGCAGAAGA
STAT3‐R	TTGTTGACGGGTCTGAAGT
Bcl‐xL‐F	GGGAGCTGGTGGTTGACTTT
Bcl‐xL‐R	GATTCAGTCCCTTCTGGGGC
Cox‐2‐F	CTTACAATGCTGACTATGGCTAC
Cox‐2‐R	ACTGATGCGTGAAGTGCTG
Cyclin D1‐F	GCGAGGAACAGAAGTGCG
Cyclin D1‐R	TGGAGTTGTCGGTGTAGATGC
C‐myc‐F	ACACCCTTCTCCCTTCG
C‐myc‐R	CCGCTCCACATACAGTCC
bcl‐2‐F	GTGGCCTTCTTTGAGTTCG
bcl‐2‐R	CCCAGCCTCCGTTATCC

#### 2.3.2. Western Blot Analysis

Total proteins were extracted using RIPA lysis buffer (Meilunbio) and quantified by BCA assay (GBCBIO). After SDS‐PAGE separation and transfer, indicated antibodies against target proteins were applied overnight at 4°C: EGFR/p‐EGFR (Immunoway, YT1497, 1:1000 dilution), STAT3/p‐STAT3 (ABclonal, AP0705, 1:1000 dilution), COX2 (Proteintech Group, 12375‐1‐AP, 1:3000 dilution), cyclin D1 (Proteintech Group, 60186‐1‐IG, 1:30,000 dilution), c‐Myc (Proteintech Group, 67447‐1‐IG, 1:30,000 dilution), BCL‐XL (Proteintech Group, 10783‐1‐AP, 1:10,000 dilution), BCL2 (Proteintech Group, 26593‐1‐AP, 1:3000 dilution), GAPDH (Servicebio, GB12002, 1:1000 dilution), and *β*‐actin (Proteintech Group, 66009‐1‐Ig, 1:10,000 dilution). The protein bands were spotted and quantified using the chemiluminescence imaging system. Quantification of band intensities was normalized to GAPDH or *β*‐actin.

#### 2.3.3. RNA Sequencing (RNA‐seq)

Total RNA was extracted using TRIzol (Invitrogen) and sent to Wuhan Linsi Biotechnology Co., Ltd. for library preparation and sequencing (Illumina platform). Differentially expressed genes (DEGs) were identified with |log2*F* *C*| ≥ 1 and *p* < 0.05.

### 2.4. Statistical Analysis

All data were analyzed using GraphPad Prism 8.0 and presented as mean ± SD. Statistical significance was determined by one‐way ANOVA with *p* < 0.05 considered statistically significant.

## 3. Results

### 3.1. Network Pharmacological Analysis and Molecular Docking

#### 3.1.1. Identification of Potential Therapeutic Targets

Bioinformatic analysis using SwissTargetPrediction database identified 64 putative molecular targets of curcumin. Concurrently, a comprehensive screening of HNSCC‐associated genes through GeneCards database initially yielded 7174 candidate genes. Following rigorous filtration to exclude targets with relevance scores < 50 and subsequent integration with two additional targets from OMIM database (after duplicate removal), we established a refined set of 1964 HNSCC‐related molecular targets.

#### 3.1.2. Determination of Shared Molecular Targets

**Figure 1:**
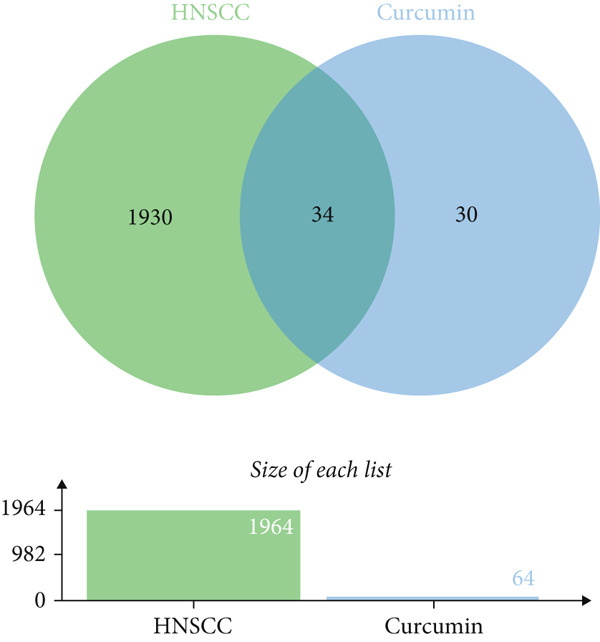
Venn diagram illustrating the overlapping targets between curcumin and HNSCC.

Intersectional analysis of the curcumin and HNSCC target sets was performed using an online Venn diagram tool, revealing 34 potential common targets (Figure 1 and Table 2). These overlapping targets represent the most promising candidates for mediating curcumin′s therapeutic effects against HNSCC.

**Table 2:** Potential therapeutic targets of curcumin in HNSCC treatment.

**Target**	**Common name**	**UniProt ID**
Epidermal growth factor receptor erbB1	EGFR	P00533
Serine/threonine–protein kinase B‐raf	BRAF	P15056
Serine/threonine–protein kinase AKT	AKT1	P31749
Signal transducer and activator of transcription 3	STAT3	P40763
Nuclear factor erythroid 2–related factor 2	NFE2L2	Q16236
Histone acetyltransferase p300	EP300	Q09472
Apoptosis regulator Bcl‐2	BCL2	P10415
Serine/threonine–protein kinase RAF	RAF1	P04049
Serine/threonine–protein kinase Chk1	CHEK1	O14757
Serine/threonine–protein kinase Aurora‐A	AURKA	O14965
Macrophage colony stimulating factor receptor	CSF1R	P07333
Beta amyloid A4 protein	APP	P05067
Matrix metalloproteinase 14	MMP14	P50281
Plasminogen activator inhibitor‐1	SERPINE1	P05121
Tyrosinase	TYR	P14679
Multidrug resistance‐associated protein 1	ABCC1	P33527
Glycogen synthase kinase‐3 beta	GSK3B	P49841
Matrix metalloproteinase 13	MMP13	P45452
ADAM17	ADAM17	P78536
Carbonic anhydrase IX	CA9	Q16790
Ribosomal protein S6 kinase 1	RPS6KB1	P23443
Serine/threonine–protein kinase WEE1	WEE1	P30291
Alkaline phosphatase, tissue‐nonspecific isozyme	ALPL	P05186
DNA topoisomerase II alpha	TOP2A	P11388
Type‐1 angiotensin II receptor (by homology)	AGTR1	P30556
Interleukin‐8 receptor B	CXCR2	P25025
Serine/threonine–protein kinase Aurora‐B	AURKB	Q96GD4
Cyclooxygenase‐1	PTGS1	P23219
DNA topoisomerase I	TOP1	P11387
5‐Lipoxygenase activating protein	ALOX5AP	P20292
Carbonic anhydrase II	CA2	P00918
Monoamine oxidase A	MAOA	P21397
Toll‐like receptor (TLR7/TLR9)	TLR9	Q9NR96
Thyroid hormone receptor beta‐1	THRB	P10828

#### 3.1.3. Network Analysis of Shared Protein Targets

**Figure 2:**
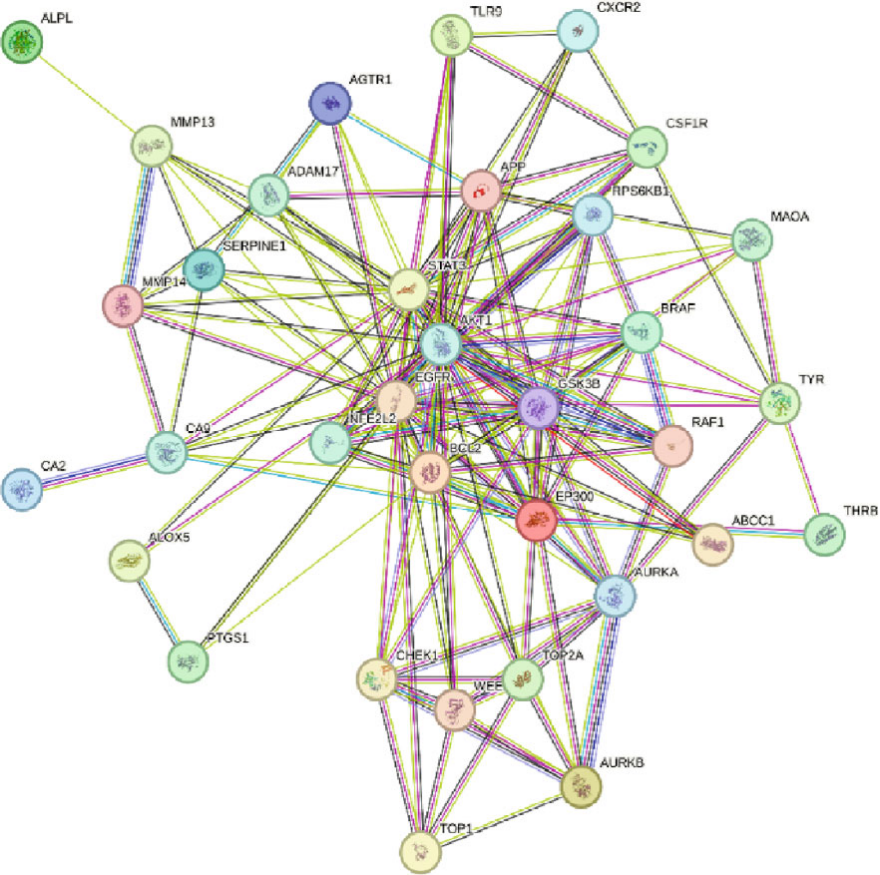
Protein–protein interaction (PPI) network of common targets between curcumin and HNSCC.

**Figure 3:**
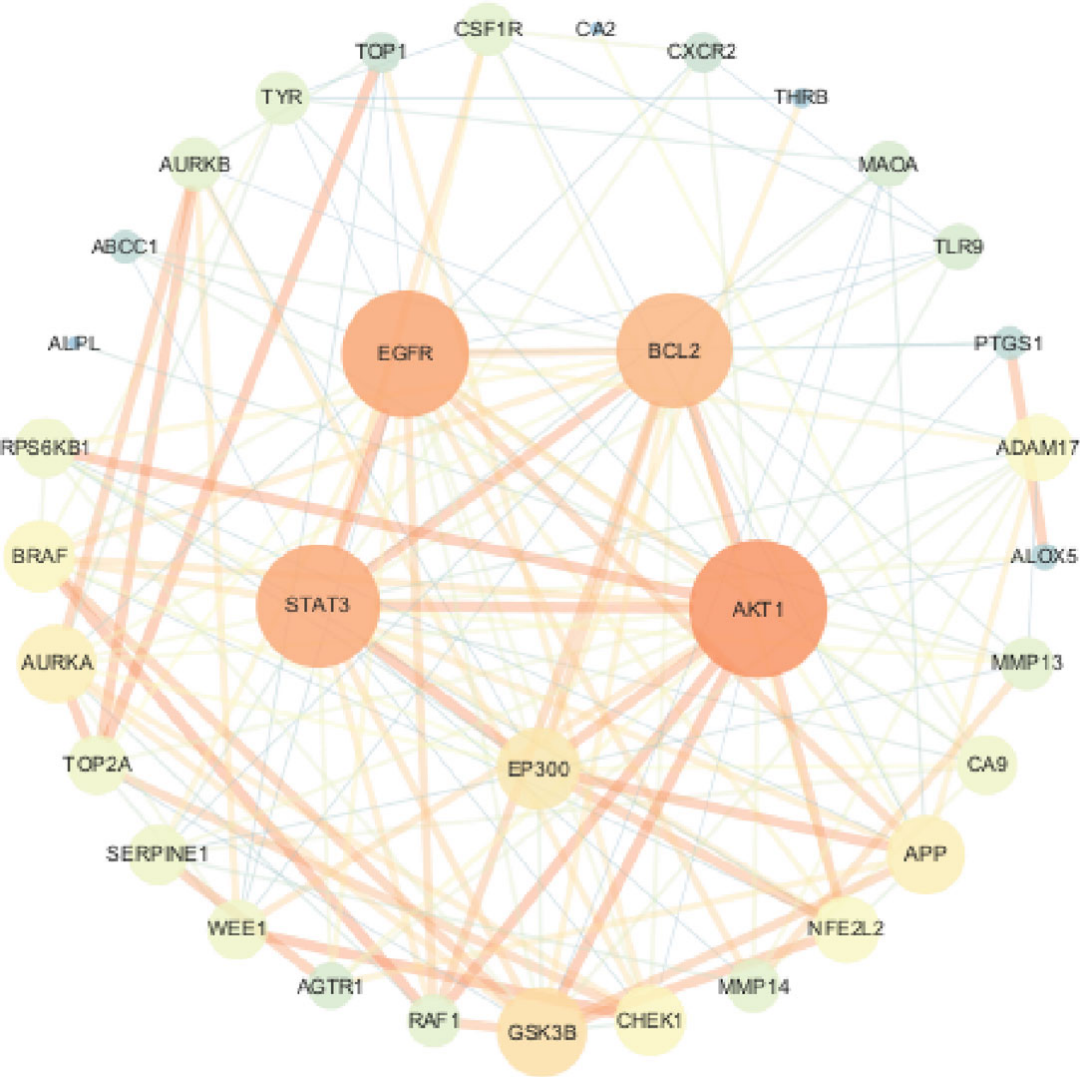
Visual representation of PPI network topology analysis.

**Figure 4:**
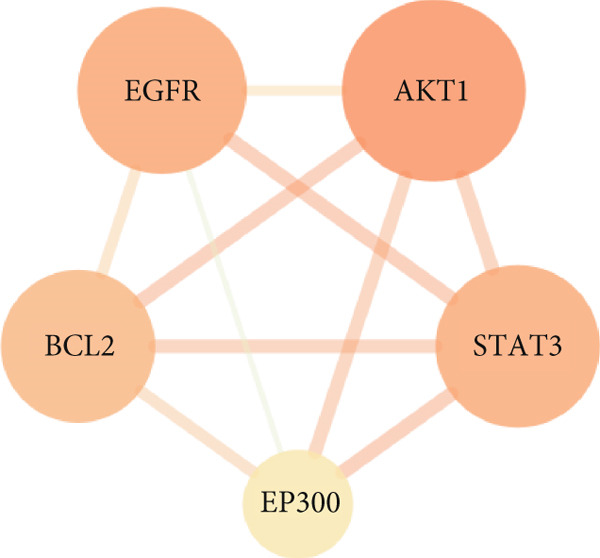
Identification of core targets in the PPI network.

The 34 overlapping targets were analyzed using the STRING database to construct a PPI network (Figure 2), consisting of 34 nodes and 157 edges, indicating curcumin′s multitarget mechanism against HNSCC. The PPI network was subsequently imported into Cytoscape 3.10.1 for topological analysis using the CentiScaPe 2.2 plugin (Figure 3). In the network visualization, node size and edge thickness were proportional to target importance, with larger nodes and thicker edges representing more significant proteins. Based on degree, closeness, and betweenness centrality values, the Top 5 hub targets were identified as AKT1, EGFR, STAT3, BCL2, and EP300 (Figure 4).

The Top 5 hub targets—AKT1, EGFR, STAT3, BCL2, and EP300—were consequently selected for subsequent molecular docking studies based on their superior performance across the three centrality measures (degree, betweenness, and closeness), which objectively signified their paramount importance within the PPI network. The EGFR/STAT3 signaling axis was further prioritized for experimental validation due to its established critical role in HNSCC oncogenesis and its central positioning within the network topology, providing a rational and efficient framework for mechanistic investigation.

#### 3.1.4. GO and KEGG Enrichment Analyses

**Figure 5:**
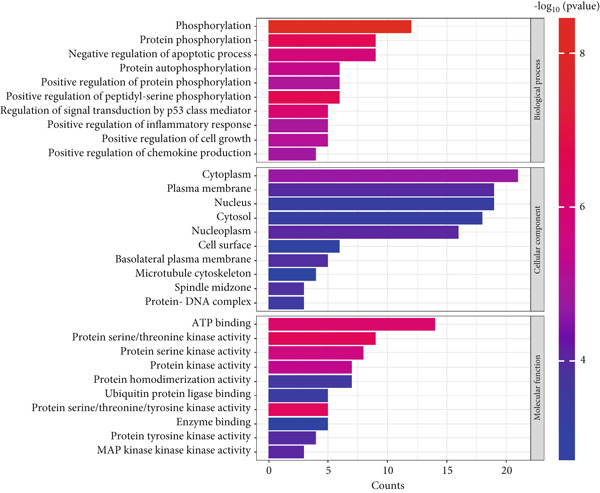
Gene Ontology (GO) enrichment analysis of shared targets.

Functional enrichment analysis was conducted on the 34 overlapping targets between curcumin and HNSCC using the DAVID database. The GO analysis yielded 149 biological process (BP) terms, 39 cellular component (CC) terms, and 42 molecular function (MF) terms. The Top 10 enriched terms from each category were selected for visualization (Figure 5). The BPs were predominantly associated with phosphorylation, positive regulation of peptidyl‐serine phosphorylation, protein phosphorylation, p53‐mediated signal transduction, and negative regulation of apoptosis. The CCs mainly included cytoplasm, nucleoplasm, plasma membrane, spindle midzone, and basolateral plasma membrane. The MFs primarily involved protein serine/threonine/tyrosine kinase activity, ATP binding, protein kinase activity, protein tyrosine kinase activity, and enzyme binding. These findings collectively suggested that curcumin may exert its effects through modulation of phosphorylation‐related processes and kinase activities in specific cellular compartments.

**Figure 6:**
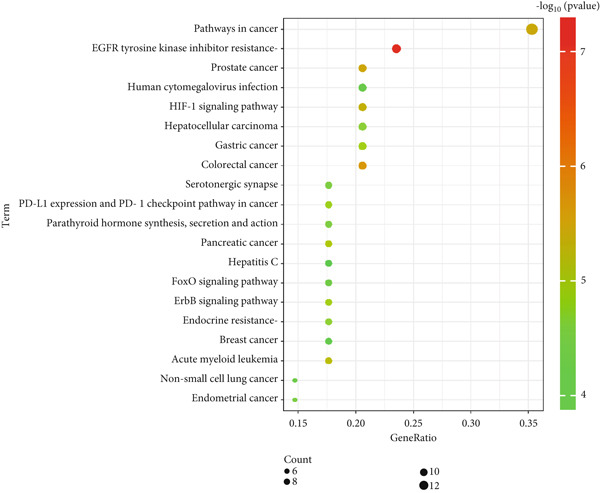
KEGG pathway enrichment analysis of shared targets.

KEGG pathway enrichment analysis of the 34 potential targets identified associations with 89 signaling pathways. The Top 20 enriched pathways were visualized in Figure 6, where the red color intensity reflects the enrichment significance and the bubble size corresponds to the number of enriched genes. The analysis indicated curcumin′s anti‐HNSCC effects primarily involve the epidermal growth factor receptor tyrosine kinase inhibitor resistance pathway, HIF‐1 signaling pathway, ErbB signaling pathway, and FoxO signaling pathway. These findings suggested the EGFR/STAT3 signaling pathway may represent a key mechanism, warranting further experimental validation based on these network pharmacology results.

#### 3.1.5. Molecular Docking Results

Molecular docking was performed using the top five core proteins (AKT1, EGFR, STAT3, BCL2, and EP300) as receptors and curcumin as the ligand. The binding energy, a critical parameter for evaluating docking efficacy, demonstrated favorable interactions with all values below −5.7 kcal/mol (Table 3). The strongest binding affinity was observed between curcumin and EGFR (−9.4 kcal/mol), while the weakest interaction occurred with AKT1 (−5.7 kcal/mol). As illustrated in Figure 7, curcumin formed stable hydrogen bonds with the target proteins, suggesting effective molecular interactions that may contribute to its pharmacological activity.

**Table 3:** Binding affinities of curcumin with core target proteins.

**Compound**	**Target**	**PDB ID**	**Binding energy (kcal/mol)**
Curcumin	STAT3	6NJS	−6.5
	EGFR	8A27	−9.4
	BCL2	8HTS	−6.3
	AKT1	7MYX	−5.7
	EP300	5BT3	−6.6

Figure 7Molecular docking of curcumin with core targets: (a) Curcumin‐STAT3 complex. (b) Curcumin‐EGFR complex. (c) Curcumin‐BCL2 complex. (d) Curcumin‐EP300 complex. (e) Curcumin‐AKT1 complex.

**Figure:**
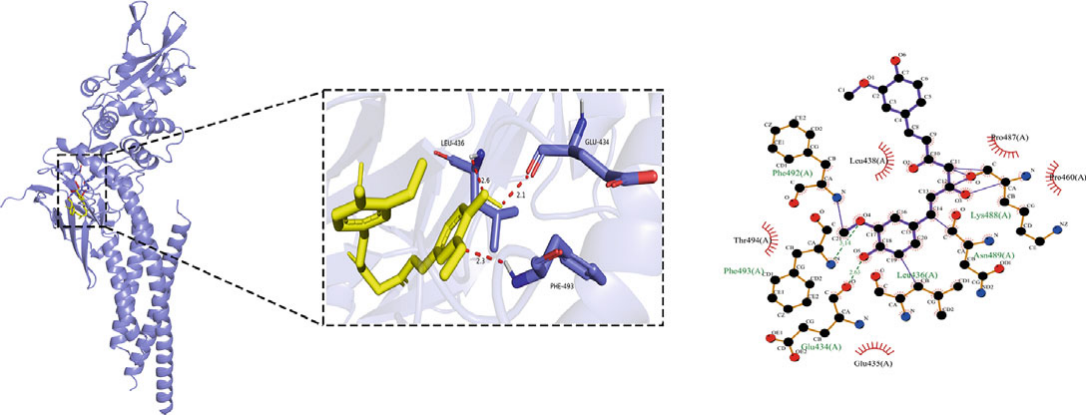
(a)

**Figure:**
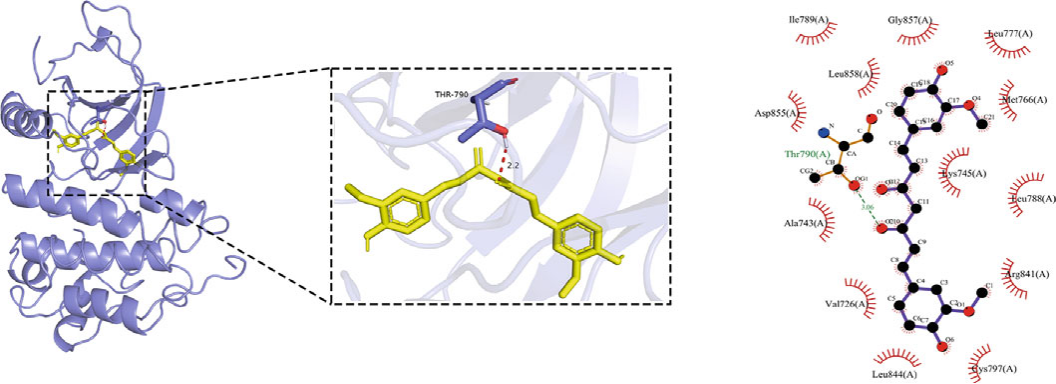
(b)

**Figure:**
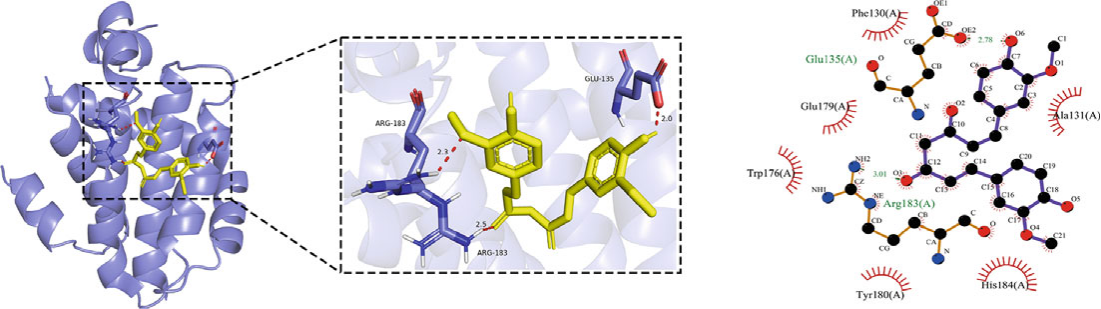
(c)

**Figure:**
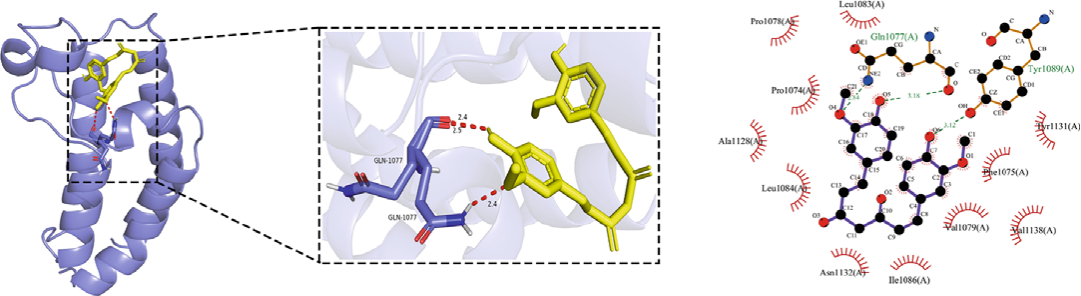
(d)

**Figure:**
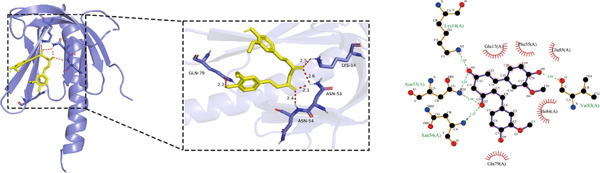
(e)

### 3.2. In Vitro Experimental Results

#### 3.2.1. Effect of Curcumin on HNSCC Cell Viability

To investigate the anti‐HNSCC activity of curcumin, we performed in vitro experiments using two HNSCC cell lines (FaDu and CAL 27). Cell viability was assessed by CCK‐8 assay, a sensitive method for measuring cell proliferation and cytotoxicity. As shown in Figure 8, treatment with 1 *μ*g/mL curcumin significantly reduced viability in both FaDu and CAL 27 cells (*p* < 0.05 and *p* < 0.01), with CAL 27 cells exhibiting greater sensitivity than FaDu cells. These results demonstrated that curcumin inhibits HNSCC cell proliferation in a dose‐dependent manner. At a 4 *μ*g/mL concentration, curcumin decreased CAL 27 cell viability to 55% while maintaining significant inhibitory effects on FaDu cells. Therefore, 4 *μ*g/mL curcumin was selected as the treatment concentration for subsequent experiments.

Figure 8CCK‐8 assay evaluating curcumin’s effect on HNSCC cell proliferation: (a) FaDu cell viability. (b) CAL27 cell viability.  ^∗^ *p* < 0.05 and  ^∗∗^ *p* < 0.01.

**Figure:**
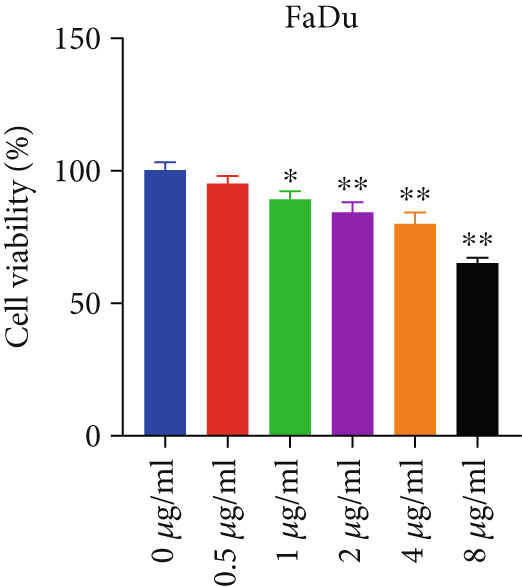
(a)

**Figure:**
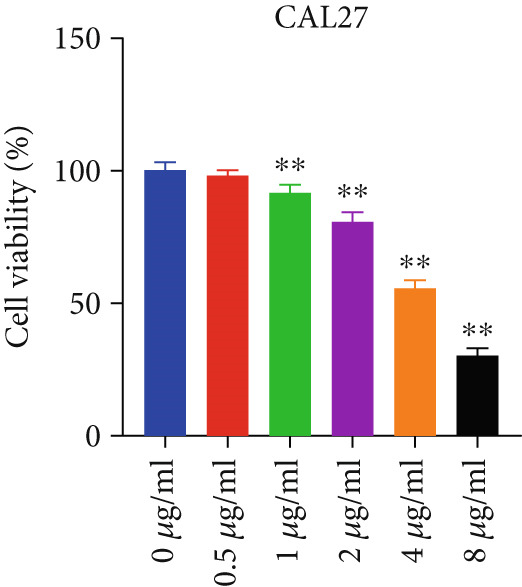
(b)

#### 3.2.2. Effect of Curcumin on HNSCC Cell Migration and Invasion

**Figure 9:**
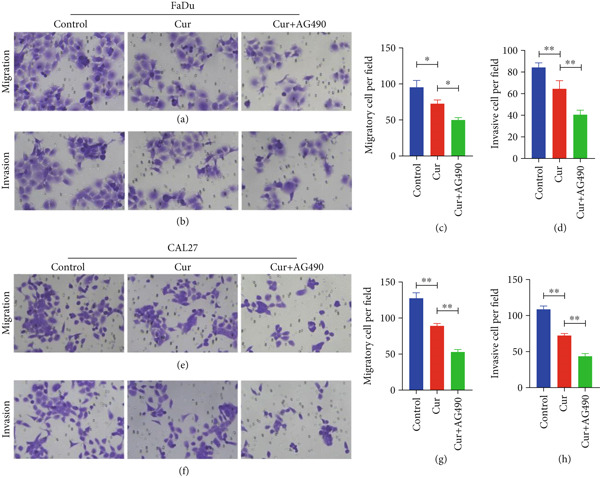
Effects of curcumin on HNSCC cell migration and invasion: (a, c) FaDu cell migration. (b, d) FaDu cell invasion. (e, g) CAL27 cell migration. (f, h) CAL27 cell invasion.  ^∗^ *p* < 0.05 and  ^∗∗^ *p* < 0.01.

Cell migration and invasion capacities were assessed by Transwell assay following 48‐h curcumin treatment. As shown in Figure 9, curcumin treatment significantly reduced the number of migrated FaDu and CAL27 cells through the membrane compared to the control group (*p* < 0.05 and *p* < 0.01), indicating marked inhibition of cell migration and invasion capabilities. Furthermore, combined treatment with the EGFR/STAT3 inhibitor AG490 resulted in additional reduction of invasive cell numbers, demonstrating enhanced inhibitory effects on cancer cell migration and invasion when curcumin was administered in combination with AG490.

#### 3.2.3. Apoptotic Effects of Curcumin on HNSCC Cells

Apoptosis was evaluated in FaDu and CAL27 cells following 48‐h treatment with varying concentrations of curcumin using Annexin V/PI double staining and flow cytometry analysis. The results demonstrated a concentration‐dependent increase in apoptotic cell populations in both cell lines (Figure 10). Comparative analysis revealed greater apoptotic rates in CAL27 cells than in FaDu cells at equivalent curcumin concentrations, indicating higher sensitivity of CAL27 cells to curcumin treatment. Based on these findings, CAL27 cells were selected for subsequent RNA‐seq analysis to examine transcriptomic changes.

Figure 10Apoptotic effects of curcumin on HNSCC cells: (a, b) Flow cytometry analysis of FaDu cell apoptosis. (c, d) Flow cytometry analysis of CAL27 cell apoptosis.  ^∗^ *p* < 0.05 and  ^∗∗^ *p* < 0.01.

**Figure:**
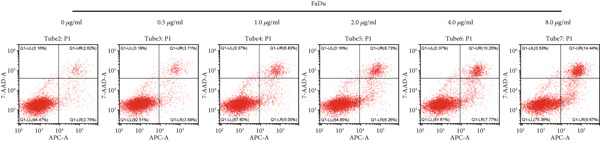
(a)

**Figure:**
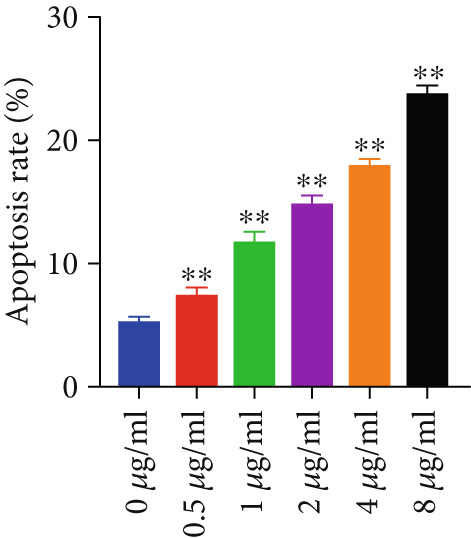
(b)

**Figure:**
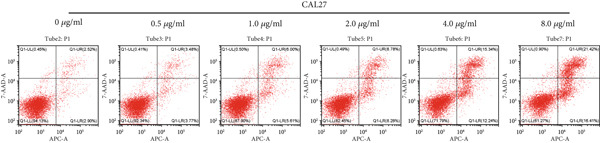
(c)

**Figure:**
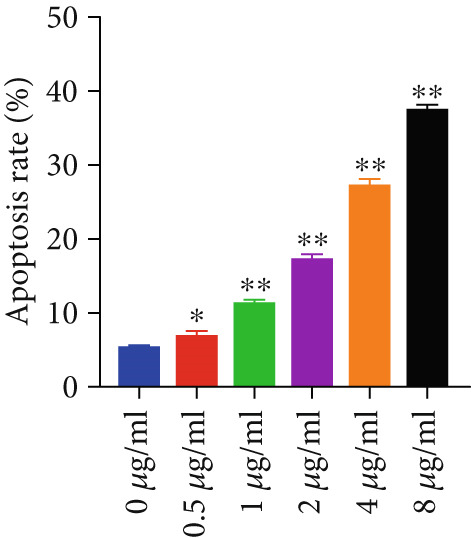
(d)

#### 3.2.4. Effect of Curcumin on EGFR/STAT3 Gene Expression in HNSCC Cells

Based on network pharmacology and molecular docking predictions identifying EGFR/STAT3 signaling as a potential key mechanism, we investigated whether curcumin modulates this pathway to affect cancer cell proliferation, apoptosis, invasion, and migration. RT‐qPCR analysis demonstrated that curcumin treatment significantly downregulated EGFR and STAT3 gene expression levels in HNSCC cells compared to controls (*p* < 0.05, Figure 11). Combined treatment with curcumin and AG490 (EGFR/STAT3 inhibitor) resulted in further reduction of EGFR and STAT3 expression levels. To further validate EGFR/STAT3 signaling pathway activity, we examined the expression of downstream target genes (COX2, Cyclin D1, c‐Myc, Bcl‐xL, and Bcl2) by qPCR. The results demonstrated that curcumin treatment markedly decreased the expression of these downstream genes in HNSCC cells (Figure 11).

Figure 11Effects of curcumin on EGFR/STAT3 and downstream target genes expression: (a) RT‐qPCR analysis in FaDu cells. (b) RT‐qPCR analysis in CAL27 cells. (c) RT‐qPCR results showed the mRNA levels of EGFR/STAT3 downstream target genes in FaDu cells. (d) RT‐qPCR results showed the mRNA levels of EGFR/STAT3 downstream target genes in CAL27 cells.  ^∗^ *p* < 0.05 and  ^∗∗^ *p* < 0.01.

**Figure:**
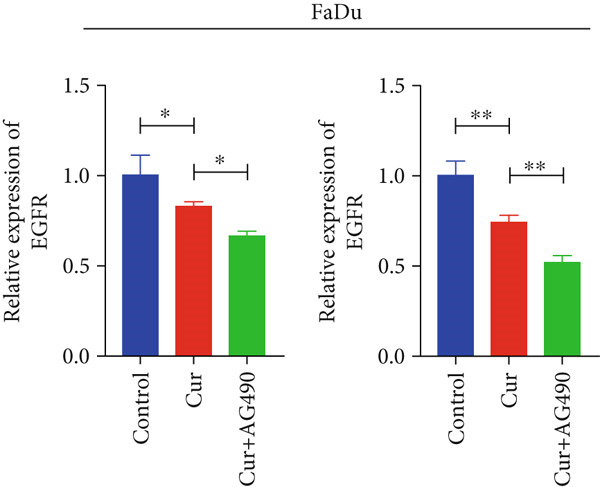
(a)

**Figure:**
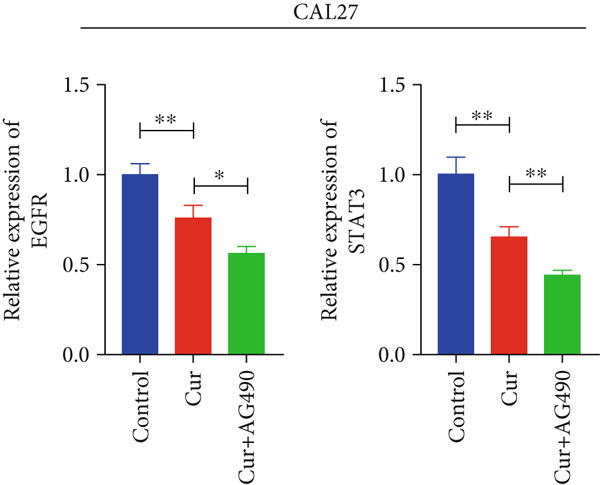
(b)

**Figure:**
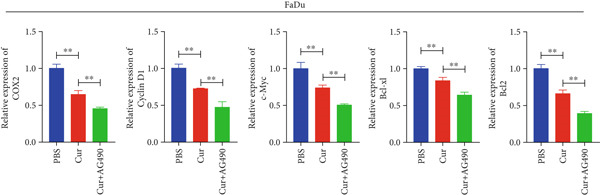
(c)

**Figure:**
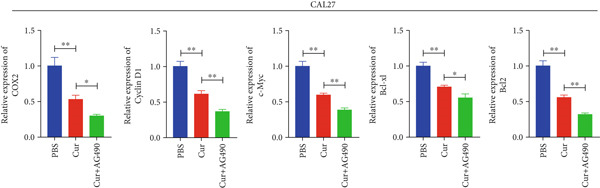
(d)

#### 3.2.5. Effect of Curcumin on EGFR/STAT3 Pathway Proteins in HNSCC Cells

The EGFR/STAT signaling pathway plays a critical role in cancer pathogenesis. Molecular docking revealed the strongest binding affinity between curcumin and EGFR, while KEGG enrichment analysis identified EGFR tyrosine kinase inhibitor resistance as the most significant pathway. Western blot analysis demonstrated that curcumin treatment significantly reduced the p‐EGFR/EGFR and p‐STAT3/STAT3 ratios in HNSCC cells compared to the controls (Figure 12). Combined treatment with curcumin and AG490 further suppressed EGFR/STAT3 pathway activity.

Figure 12Effects of curcumin on EGFR/STAT3 signaling pathway: (a–c) Western blot analysis in FaDu cells. (d–f) Western blot analysis in CAL27 cells. (g–h) The expressions of COX2, Cyclin D1, c‐Myc, Bcl‐xL, and Bcl2 in FaDu cells were analyzed using western blot analysis. (i, j) The expressions of COX2, Cyclin D1, c‐Myc, Bcl‐xL, and Bcl2 in CAL27 cells were analyzed using western blot analysis.  ^∗^ *p* < 0.05 and  ^∗∗^ *p* < 0.01.

**Figure:**
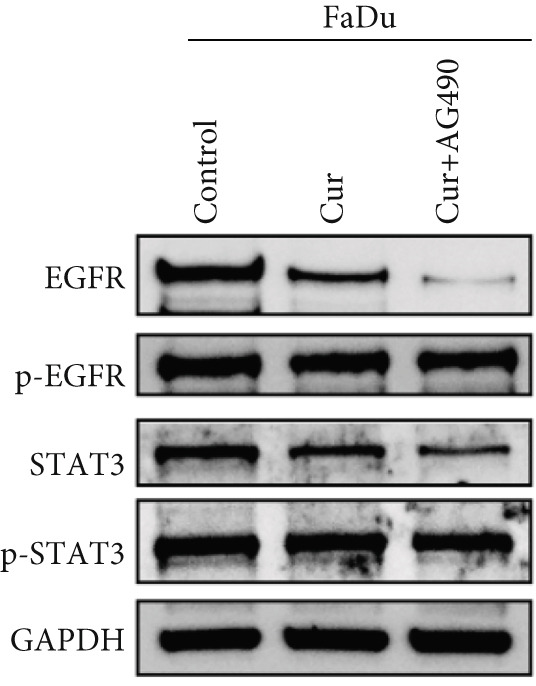
(a)

**Figure:**
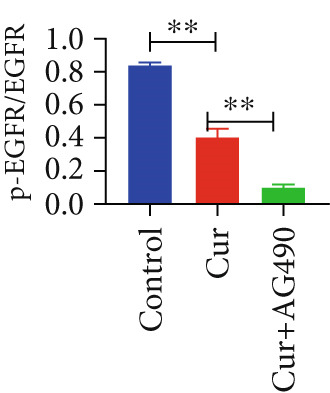
(b)

**Figure:**
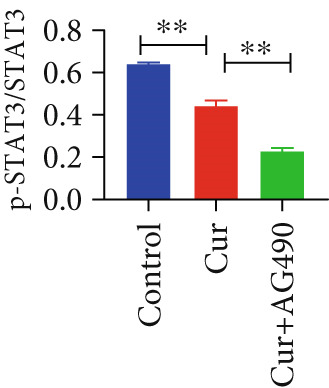
(c)

**Figure:**
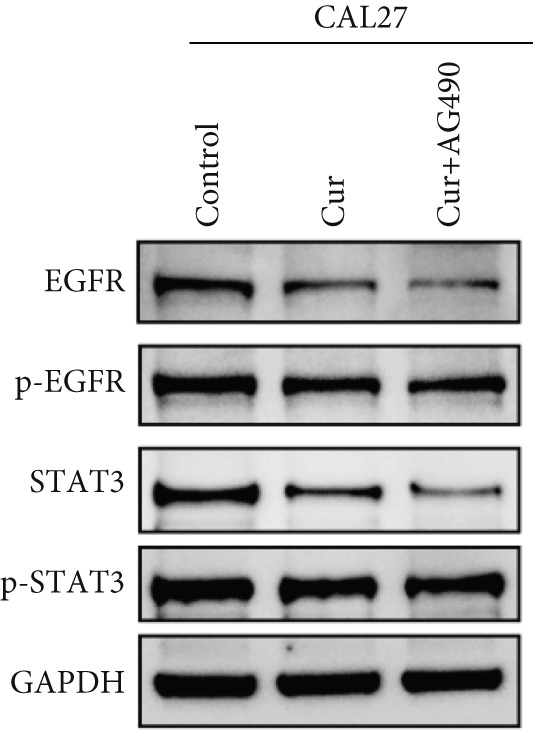
(d)

**Figure:**
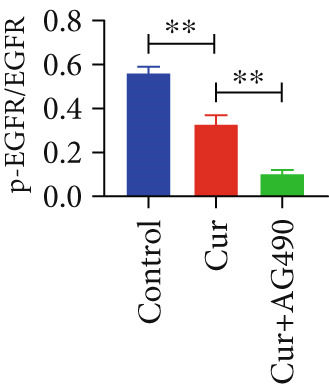
(e)

**Figure:**
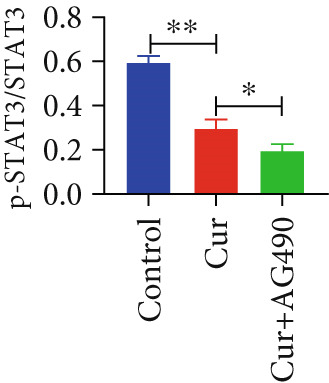
(f)

**Figure:**
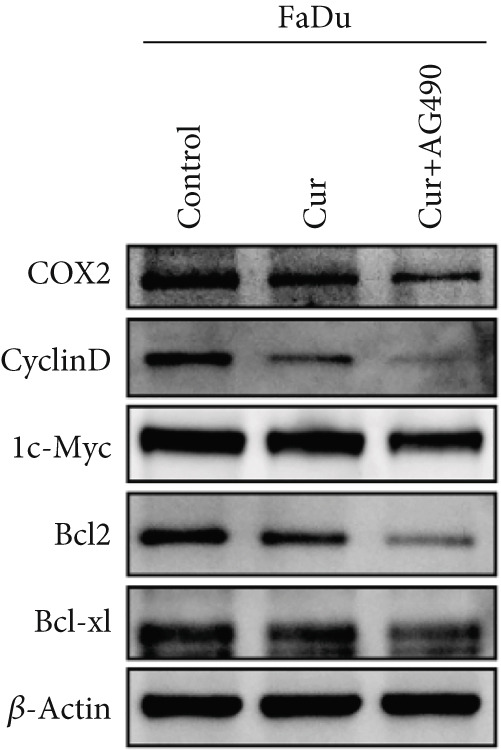
(g)

**Figure:**
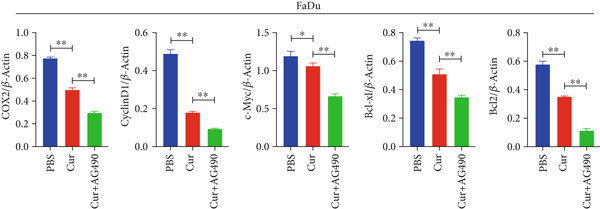
(h)

**Figure:**
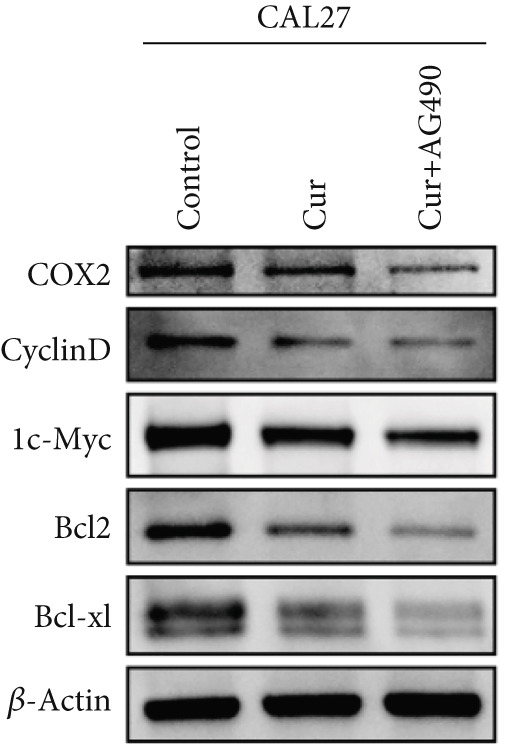
(i)

**Figure:**
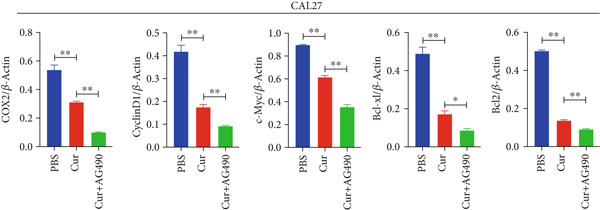
(j)

Further western blot analysis of downstream effector proteins confirmed that curcumin significantly decreased the expression of key regulatory proteins (*p* < 0.05; Figure 12). These results collectively indicate that curcumin modulates cancer cell proliferation, apoptosis, invasion, and migration through inhibition of the EGFR/STAT3 signaling pathway.

#### 3.2.6. Transcriptomic Effects of Curcumin on HNSCC Cells

CAL27 cells were divided into control (CK) and curcumin‐treated (Cur) groups, with three biological replicates per group. Principal component analysis (PCA) was performed based on the first (PC1) and second (PC2) principal components of gene expression variation. The PCA plot demonstrated minimal intragroup variation but significant intergroup separation between CK and Cur samples, indicating good clustering efficiency within each group (Figure 13a). This clustering pattern was further validated by correlation analysis (Figure 13b).

Figure 13Transcriptomic analysis: (a) Principal component analysis. (b) Correlation analysis.

**Figure:**
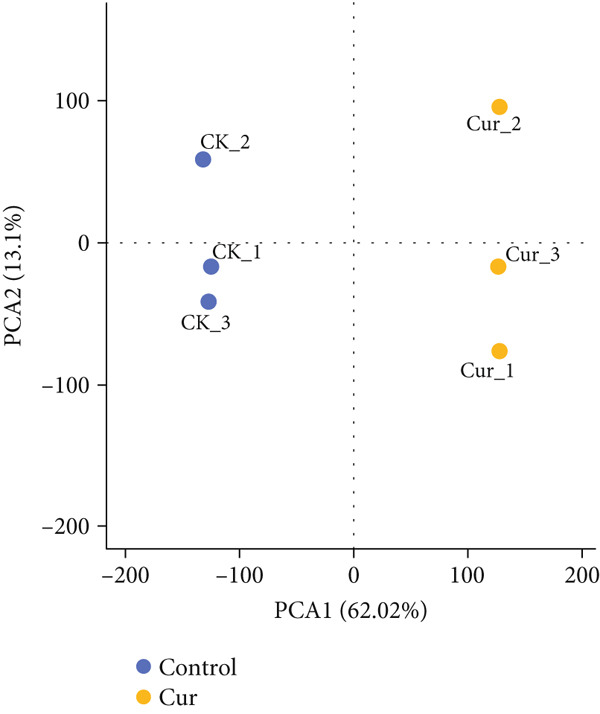
(a)

**Figure:**
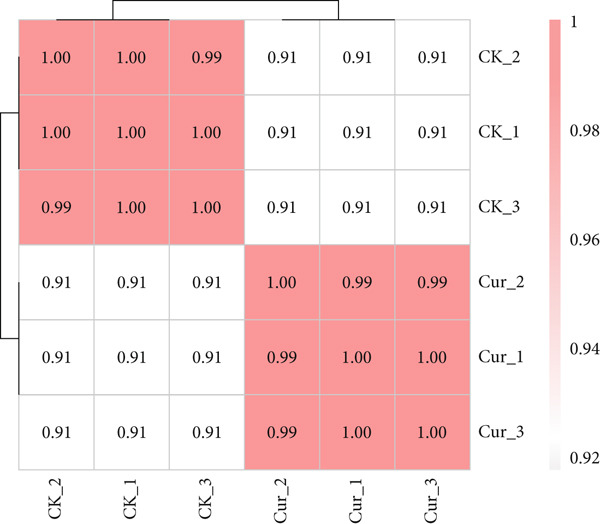
(b)

Comparative analysis of CK and Cur groups identified DEGs using thresholds of FC > 3.0 and *p* < 0.05. A total of 5411 DEGs were detected, comprising 4570 upregulated and 841 downregulated genes. The differential expression patterns were visualized in the volcano plot (Figure 14a) and heat map (Figure 14b), where blue dots represent downregulated genes, red dots indicate upregulated genes, and gray dots denote nonsignificant changes. Notably, nine genes exhibited particularly strong upregulation (> 11‐fold change): PDE4C, SCUBE2, LOC100506358, LOC107985256, LOC105375038, PTGER3, AMPD1, LOC105371634, and ARHGAP15.

Figure 14Differential gene expression analysis: (a) Volcano plot. (b) Heatmap.

**Figure:**
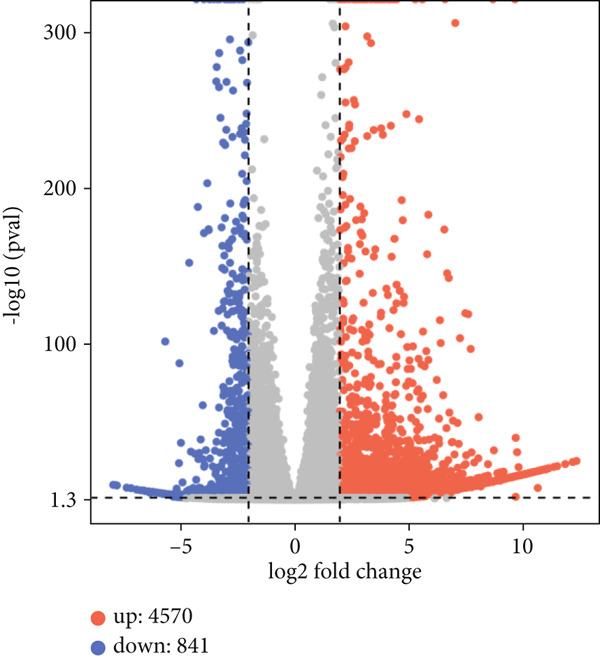
(a)

**Figure:**
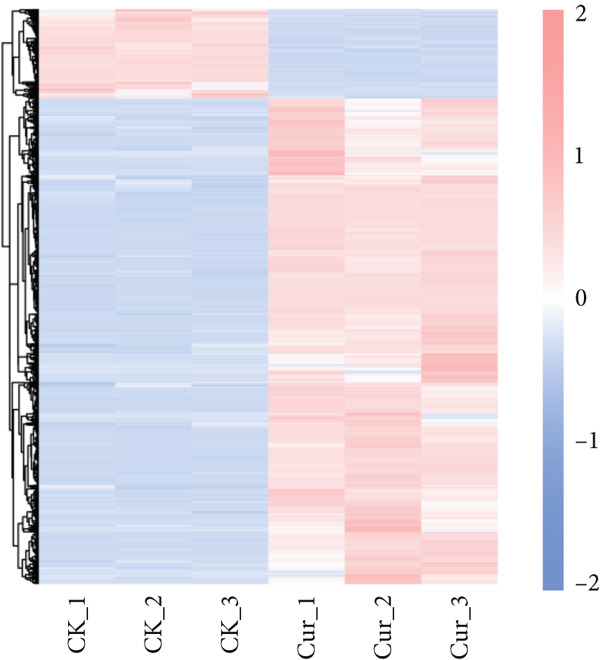
(b)

**Figure 15:**
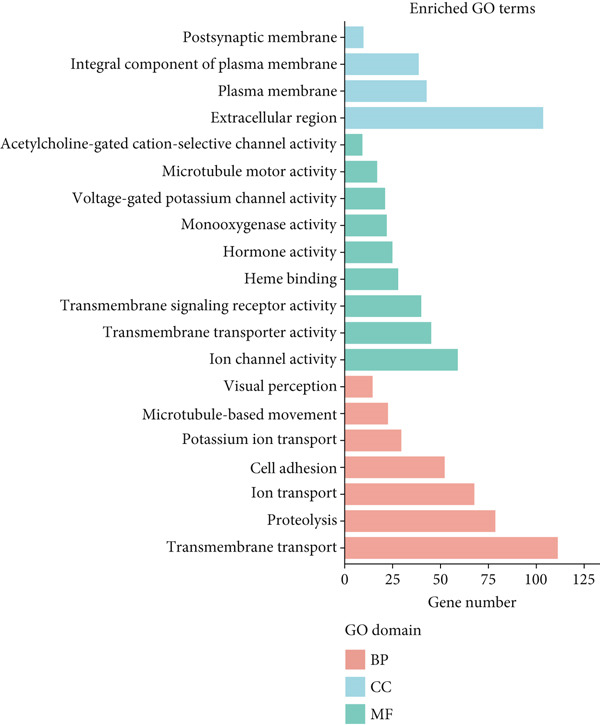
GO enrichment analysis of differentially expressed genes.

GO enrichment analysis of DEGs between CK and Cur groups identified 81 significant functional terms (Figure 15), including 30 BPs, 10 CCs, and 41 MFs. The Top 10 enriched functional categories were as follows: ion transport, ion channel activity, transmembrane transport, extracellular region, transmembrane signaling receptor activity, potassium ion transport, transmembrane transporter activity, voltage‐gated potassium channel activity, hormone activity, and integral component of plasma membrane. Notably, five of these Top 10 functional categories belonged to the MF group.

**Figure 16:**
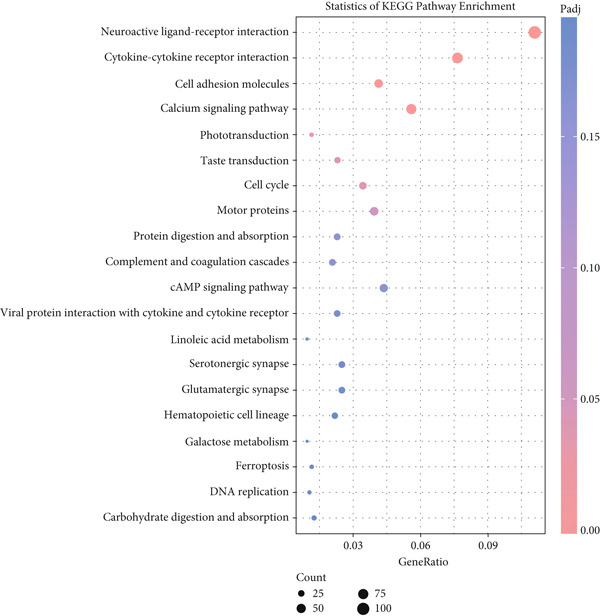
KEGG pathway enrichment analysis of differentially expressed genes.

KEGG pathway enrichment analysis was performed on DEGs between the CK and Cur groups (Figure 16). In the visualization plot, the vertical axis displays pathway information while the horizontal axis represents the rich factor, defined as the ratio of DEGs annotated to a given pathway versus all genes annotated to that pathway. The rich factor value positively correlates with functional significance, with higher values indicating greater enrichment significance of DEGs in the pathway. Dot color represents the *p* value, where smaller *p* values indicate more reliable enrichment significance, and dot size corresponds to the number of enriched genes in each pathway.

The analysis identified 33 significantly enriched KEGG pathways. The most prominent pathway was the neuroactive ligand–receptor interaction pathway, containing 107 enriched DEGs. The JAK‐STAT signaling pathway showed enrichment of 30 DEGs.

Our screening identified 153 STAT pathway‐related genes (Figure 17a). Among DEGs, we detected 30 STAT pathway‐associated genes (Figure 17b). Venn diagram analysis (Figure 17c) revealed that all STAT pathway–related DEGs, except IL22RA1, showed significant downregulation following curcumin treatment. These results demonstrated curcumin′s pronounced regulatory effect on the STAT pathway.

Figure 17STAT pathway‐related gene analysis: (a) Expression profiles of STAT pathway genes. (b) Differentially expressed STAT pathway genes. (c) Venn diagram of differential genes and STAT pathway–related genes.

**Figure:**
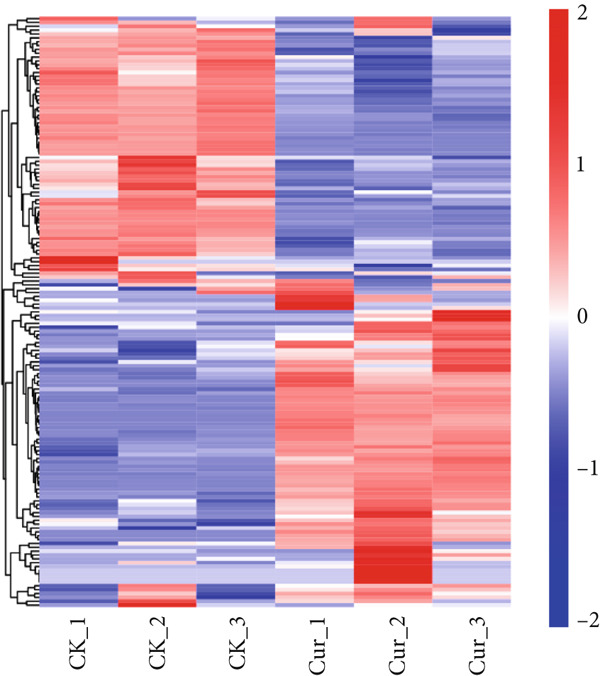
(a)

**Figure:**
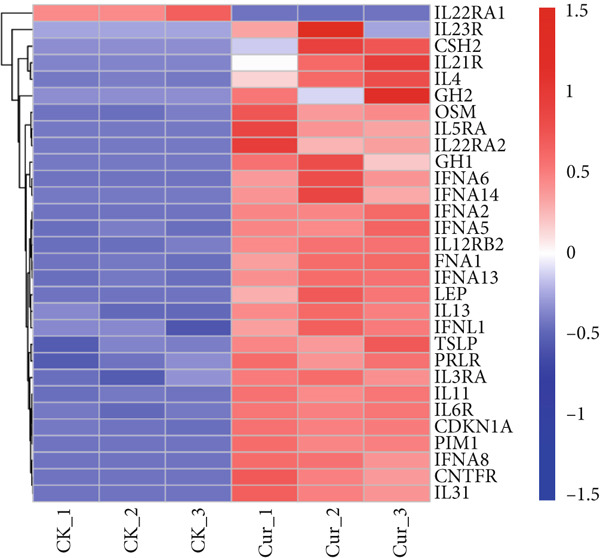
(b)

**Figure:**
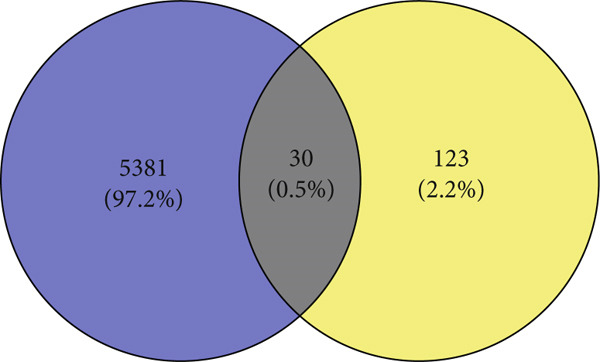
(c)

## 4. Discussion

### 4.1. Significance and Innovation of Network Pharmacology in Studying Curcumin’s Anti‐HNSCC Effects

HNSCC represents a highly heterogeneous malignancy with approximately 650,000 new cases and 330,000 deaths annually worldwide, showing an increasing incidence trend [22]. While smoking and alcohol remain primary risk factors, regional variations exist with betel nut chewing and HPV infection contributing to disease prevalence [23]. Current treatment modalities, including surgery, chemotherapy, radiotherapy, and monoclonal antibodies, face challenges such as local tissue destruction, high recurrence rates, and functional impairments. These limitations underscore the urgent need for developing novel therapeutic strategies, combining modern treatments with TCM compounds demonstrating efficacy and low toxicity.

Natural plant–derived compounds have emerged as promising anticancer agents. Paclitaxel, camptothecin, and vincristine exemplify successful plant‐based chemotherapeutics [24]. Curcumin, a phenolic compound from turmeric, has gained attention for its multitarget anticancer properties validated in clinical trials. Network pharmacology integrates multidisciplinary approaches to elucidate complex drug–target–disease relationships, providing systematic insights into traditional medicine mechanisms. Our study represented the first comprehensive investigation combining network pharmacology and molecular docking to identify curcumin′s potential targets and mechanisms against HNSCC.

### 4.2. Core Targets of Curcumin in HNSCC

Topological analysis identified AKT1, EGFR, STAT3, BCL2, and EP300 as central targets in curcumin′s anti‐HNSCC network, with molecular docking confirming strong binding affinities.

AKT1, a serine/threonine kinase regulating cell growth and survival pathways, demonstrates frequent overexpression in cancers [25, 26]. Previous studies have shown that curcumin modulates PI3K/AKT/mTOR signaling in hypopharyngeal and tongue carcinomas [27, 28]. EGFR, overexpressed in HNSCC, activates multiple oncogenic pathways including PI3K‐AKT and JAK‐STAT [29, 30]. Despite EGFR‐targeted therapies like cetuximab, resistance remains a clinical challenge [31, 32].

STAT3 mediates critical cancer processes through transcriptional regulation of survival genes [33]. Its constitutive activation correlates with tumor progression across malignancies [34–36]. The BCL2 protein family governs mitochondrial apoptosis, with antiapoptotic members frequently overexpressed in cancers [37–40]. EP300, a histone acetyltransferase, regulates gene expression and has been implicated in oral squamous cell carcinoma progression through TGF‐*β*/Smad4 signaling [41–43].

These targets form an interconnected network: AKT1 and EGFR share PI3K pathway components; STAT3 regulates BCL2 expression; and EP300 mediates epigenetic modifications. This multidimensional interaction pattern has provided theoretical support for developing curcumin‐based combination therapies.

### 4.3. Curcumin Inhibits Proliferation, Invasion, and Migration While Inducing Apoptosis in HNSCC Cells

Curcumin has demonstrated potential for treating various diseases, including cancer, and is recognized as a multitarget agent with antitumor activity. To date, extensive research has been conducted on its potential in vitro anticancer effects. In this study, we investigated the cytotoxic effects of curcumin on FaDu and CAL 27 cells through cellular experiments.

Apoptosis, a programmed cell death process, plays a crucial role in maintaining cellular homeostasis [44]. However, cancer cells can evade this self‐destruction mechanism by overexpressing specific proteins that activate or inhibit related apoptotic signaling pathways, leading to uncontrolled proliferation. To evaluate the effect of curcumin on cancer cell proliferation, we performed CCK‐8 assays. The results revealed that curcumin exhibited significant cytotoxicity, markedly inhibiting the proliferation of FaDu and CAL 27 cells in a dose‐dependent manner compared to the control group.

Cancer metastasis is a multistep process involving migration and invasion into surrounding tissues, whereby tumor cells disseminate from the primary site to various parts of the body, ultimately causing multiorgan failure, which accounts for the majority of cancer‐related deaths [45]. Invasion is considered the initial phase of metastasis, during which cancer cells penetrate the basement membrane and migrate into adjacent tissues. In this study, Transwell assays further demonstrated that curcumin treatment significantly reduced the invasive and migratory capabilities of cancer cells. These findings are consistent with previous studies reporting that curcumin inhibits migration and invasion in pancreatic cancer and bladder cancer cells [46, 47]. Notably, the combination of curcumin with AG490 enhanced its inhibitory effects on cancer cell migration and invasion.

Finally, flow cytometry with double staining confirmed that curcumin promoted apoptosis in FaDu and CAL 27 cells in a concentration‐dependent manner in vitro. Collectively, our results demonstrate that curcumin exerts its antitumor effects by suppressing cancer cell proliferation, invasion, and migration, while simultaneously inducing apoptosis. These findings provide a solid experimental foundation for the potential application of curcumin in HNSCC treatment.

### 4.4. Curcumin Suppresses EGFR/STAT3 Signaling Pathway

Molecular docking results indicated that curcumin exhibited the most favorable binding energy and a stable docking conformation at the active site of EGFR. Additionally, the predicted binding affinity for STAT3 was superior to that for BCL2. KEGG pathway enrichment analysis further revealed that the “EGFR tyrosine kinase inhibitor resistance” pathway was the most significantly enriched. In cancer cells, blockade of the JAK/STAT signaling pathway can suppress the expression of target genes that regulate fundamental cellular processes, while simultaneously inhibiting apoptosis and invasion of cancer cells. Current evidence has suggested that persistent STAT3 nuclear translocation and consequent hyperactivation are closely associated with HNSCC pathogenesis. Previous studies have demonstrated that curcumin can reduce STAT3 levels and inhibit both STAT3 phosphorylation and nuclear translocation. For instance, Abdolahinia et al. [48] have shown in a study using the HN5 HNSCC line that curcumin increased Caspase‐9 expression and the Bax/Bcl‐2 ratio while decreasing STAT3 expression levels, thereby promoting apoptosis. Furthermore, phosphorylated EGFR can activate downstream signaling cascades, with STAT3 serving as one of the key mediators in this process [49].

Based on network pharmacology GO functional annotation and KEGG pathway analysis, we predicted that the EGFR/STAT3 signaling pathway might represent one of the key mechanisms underlying curcumin′s effects. Consequently, we examined the impact of curcumin on EGFR and STAT3 expression at both gene and protein levels in FaDu and CAL 27 cells. Our results demonstrated that curcumin treatment significantly downregulated EGFR and STAT3 gene expression while reducing p‐EGFR and p‐STAT3 protein levels. These findings suggested that curcumin exerts its anti‐HNSCC effects through suppression of EGFR/STAT3 pathway activation. Therefore, our study not only validated a novel molecular mechanism involved in HNSCC progression but also identified a potential therapeutic target for HNSCC treatment.

### 4.5. Transcriptomic Insights Into Curcumin’s Mechanisms

RNA‐seq is currently recognized as the most robust, reliable, and versatile technology for genome‐wide measurement of gene expression and transcriptional activation. With its capability to identify novel genes and detect allele‐specific expression patterns, RNA‐seq has become an indispensable tool in pharmacological mechanism research [50, 51]. Leveraging high‐throughput sequencing‐based transcriptomic profiling platforms and advanced bioinformatic analysis approaches, we can now systematically identify cancer biomarkers, genetic signatures, and dysregulated expression patterns during tumorigenesis. This technological advancement has opened new avenues for discovering molecular targets in anticancer therapy.

RNA‐seq analysis revealed significant transcriptomic alterations in CAL27 cells following curcumin treatment, with 5411 DEGs (4570 upregulated and 841 downregulated). Notably, 29 of 30 STAT pathway‐related DEGs showed downregulation, excepting IL22RA1. These findings have systematically characterized curcumin′s gene regulatory effects, particularly its suppression of STAT signaling.

The convergence of network pharmacology predictions and transcriptomic validations exemplified a systems biology approach, combining topological network analysis with genome‐wide expression profiling [52]. This integrative strategy advances precision medicine by elucidating complex therapeutic mechanisms while identifying potential intervention targets for HNSCC treatment.

## 5. Conclusions

This study systematically elucidated the molecular mechanisms underlying the anti‐HNSCC effects of curcumin through an integrated approach combining network pharmacology, molecular docking, and in vitro experiments. Network pharmacological analysis identified AKT1, EGFR, STAT3, BCL2, and EP300 as core targets of curcumin, with the EGFR/STAT3 signaling pathway being identified as a crucial regulatory axis. In vitro experiments demonstrated that curcumin significantly inhibited the proliferation, invasion, and migration of HNSCC cells (FaDu and CAL27), while promoting apoptosis. At the molecular level, curcumin downregulated the gene expression of EGFR and STAT3, reduced the p‐EGFR/EGFR and p‐STAT3/STAT3 protein ratios, and effectively suppressed the EGFR/STAT3 signaling pathway. Transcriptomic analysis further revealed that curcumin markedly altered the gene expression profile of CAL27 cells and exerted its antitumor effects by modulating the STAT pathway. These findings provided novel theoretical insights into the therapeutic potential of curcumin for HNSCC and highlight its promise as a potential therapeutic agent. While this study provides preliminary data and insights into the antitumor effects of curcumin against HNSCC, it is not without limitations, one of which pertains to the selection of cell models. All in vitro conclusions are based on experimental results obtained from only two HNSCC cell lines (FaDu and CAL27). Although this approach ensured internal consistency and reproducibility, it also limits the generalizability of the findings. Specifically, FaDu and CAL27 represent only certain molecular subtypes and phenotypes of HNSCC and cannot fully capture the extensive heterogeneity of the disease. Further studies are warranted to evaluate its efficacy in animal models and clinical settings.

**Nomenclature**
CUR: curcumin
HNSCC: head and neck squamous cell carcinoma
PBS: phosphate‐buffered saline
PPI: protein–protein interaction network
GO: Gene Ontology
BP: biological process
CC: cellular component
MF: molecular function
RT‐qPCR: real‐time quantitative polymerase chain reaction
PVDF: polyvinylidene fluoride
OD: optical density
WB: western blot
RNA‐Seq: RNA sequencing
FC: fold change
PC: principal component
EGFR: epidermal growth factor receptor
STAT3: signal transducer and activator of transcription‐3
KEGG: Kyoto Encyclopedia of Genes and Genomes

## Conflicts of Interest

The authors declare no conflicts of interest.

## Author Contributions

Yating He and Yaqi Liao contributed equally.

## Funding

This work was supported by the Hubei Provincial Health Commission Scientific Research Project, WJ2021F143.

## General Statement

*Copyright*. 2025 Yating He et al. BioMed Research International published by John Wiley & Sons Ltd. This is an open access article under the terms of the Creative Commons Attribution License, which permits use, distribution, and reproduction in any medium, provided the original work is properly cited.

## Data Availability

The data that support the findings of this study are available from the corresponding author upon reasonable request.
